# Correlated responses to clonal selection in populations of *Daphnia pulicaria*: mechanisms of genetic correlation and the creative power of sex

**DOI:** 10.1002/ece3.444

**Published:** 2012-12-19

**Authors:** Jeffry L Dudycha, Margaret Snoke-Smith, Ricardo Alía

**Affiliations:** 1 Department of Biological Sciences, University of South CarolinaColumbia, SC, 29208; 2 Department of Biology, North Georgia College & State University, Health & Natural SciencesBuilding, 322 Sunset Dr., Dahlonega, GA, 30597; 3 Instituto Nacional de Investigación y Tecnología Agraria y AlimentariaCrta. de la Coruña, km 7,5, 28040, Madrid, Spain

**Keywords:** Body size, complex traits, life history, linkage, pleiotropy, recombination

## Abstract

Genetic correlations among traits alter evolutionary trajectories due to indirect selection. Pleiotropy, chance linkage, and selection can all lead to genetic correlations, but have different consequences for phenotypic evolution. We sought to assess the mechanisms contributing to correlations with size at maturity in the cyclic parthenogen *Daphnia pulicaria*. We selected on size in each of four populations that differ in the frequency of sex, and evaluated correlated responses in a life table. Size at advanced adulthood, reproductive output, and adult growth rate clearly showed greater responses in high-sex populations, with a similar pattern in neonate size and *r*. This pattern is expected only when trait correlations are favored by selection and the frequency of sex favors the creation and demographic expansion of highly fit clones. Juvenile growth and age at maturity did not diverge consistently. The inter-clutch interval appeared to respond more strongly in low-sex populations, but this was not statistically significant. Our data support the hypothesis that correlated selection is the strongest driver of genetic correlations, and suggest that in organisms with both sexual and asexual reproduction, adaptation can be enhanced by recombination.

## Introduction

Genetic correlations among traits can alter the evolutionary trajectory of a trait across the adaptive landscape because traits are indirectly subjected to the selection pressures aimed at correlated traits (Maynard Smith et al. 1985; Falconer and Mackay 1996). Drawing on the idea that linked genes can interfere with the most rapid fixation of beneficial traits, correlated traits are often thought of as impeding the evolutionary approach to an optimum trait value (Lande 1979; Clark 1987; Mitchell-Olds 1996; Roff and Fairbairn 2006). However, if both traits are advantageous under the same circumstances, genetic correlations can potentially accelerate adaptation. Such synergistic selection is thought to lead to the erasure of the correlation as genetic variation declines (Falconer and Mackay 1996), but such correlations may be observed prior to fixation. Furthermore, it is possible for a correlation itself to have adaptive value; that is, the fitness of a particular value of one trait may depend on the value of another trait (Brodie 1992; Sinervo and Svensson 2002).

The consequences genetic correlations can have for phenotypic evolution have driven interest in the mechanisms leading to genetic correlations. Traits can be correlated for multiple reasons, including pleiotropy, linkage due to chance, or linkage due to selection (Houle 1991; Armbruster and Schwaegerle 1996; Lynch and Walsh 1998; Roff and Fairbairn 2006). Pleiotropy results in correlations between traits because the same gene(s) underlie the correlated traits (Lande 1980; McKay et al. 2003). For example, a single gene can be an upstream member of multiple genetic pathways, with allelic variation in that gene or its regulation leading to consequences in distinct downstream outcomes. Correlations between traits can also occur by chance if genes controlling the traits are physically linked in the genome (Lande 1976), limiting the dissolution of allelic associations by recombination. Lastly, traits can be correlated if there is selection for the maintenance of a suite of phenotypic traits (Lande 1984). Determining the extent to which these different mechanisms contribute to trait correlations is an important foundation for understanding the evolution of complex traits.

Drawing on classic work by Fisher (1958) and Maynard Smith (1978), evolutionary biologists have been fascinated by recombination because of its apparent costliness and potential to change the dynamics of adaptation (reviewed in West et al. 1999; Lehtonen et al. 2012; Meirmans et al. 2012). Recombination influences genetic correlations among traits differently depending on the mechanism that leads to correlations. Both the chance correlations due to physical linkage and adaptive correlations exist in a state of linkage disequilibrium. If recombination is frequent enough in populations with standing genetic variation for the traits in question, both will consequently be limited. Theoretical and empirical work has shown that recombination breaks up physically linked genes helping to eliminate interference via indirect effects of selection (Falconer and Mackay 1996; Colegrave 2002). These two mechanisms differ in that under physical linkage, correlations will be transitory and are not expected to have any consistency across ecologically similar populations. In contrast, correlations driven by selection will exist as a balance between recombination and selection, and should take the same basic structure across ecologically similar populations. Unlike correlations arising from disequilibrium, correlations arising from pleiotropy will exist irrespective of recombination and selection.

Work by Baer and Lynch (2003) provides a framework for differentiating between correlations maintained by different mechanisms. By looking at the correlations that appear within and between populations, they distinguished between pleiotropy and selection. This was done by assaying body size on clones from two populations of *Daphnia* in a laboratory common garden, and then selecting the largest and smallest clones for a subsequent life history assay. Baer and Lynch (2003) expected a similar pattern of correlation between large and small body size within populations to that found between populations if correlations were maintained because of pleiotropy. However, they concluded that selection was maintaining correlations between traits in their populations because correlation patterns within the two populations they studied differed from the trait associations between the two populations' trait averages.

**Figure 1:**
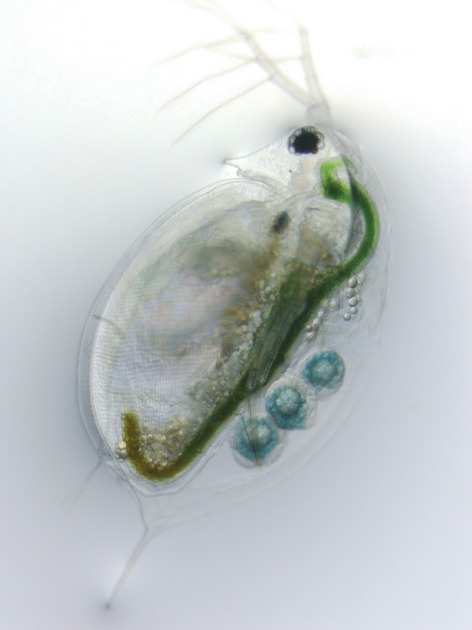
*Daphnia pulicaria*. Photograph courtesy Christine Ansell.

Here, we apply an analogous approach by examining correlated responses to clonal selection on body size in four different populations of *Daphnia pulicaria* (Fig. 1). We focus on ecologically similar populations with historically different levels of sex (Cáceres and Tessier 2004). This design allows us to explore how recombination affects the response to direct and indirect selection via genetic correlations and evaluate the potential mechanisms for how these correlations are being maintained. First, we examine the direct response of body size to clonal selection for large and small body size at maturity. Second, we examine correlated responses in life history traits in the selected clones in the context of differences in historical frequencies of sex. In particular, we ask (1) whether historical frequency of sex influences the magnitude of correlated responses to clonal selection; and (2) whether the slope of genetic correlations is uniform across populations.

### Predicted correlated responses

The mechanisms which cause correlations to evolve lead to contrasting predictions in our system. First, consider the distinction between pleiotropy and linkage-based mechanisms. If genetic correlations are driven by pleiotropies, we predict that correlated responses will be unaffected by the historical frequency of sex. If this were true, and trait correlations were governed by pleiotropies of the same set of genes drawing on a common pool of allelic variation, we would expect the slope of the association to be similar across populations. If, however, the association between two traits was driven by allelic variation at one pleiotropic gene (or set of genes) in one population, and by allelic variation at different pleiotropic genes in another population, the slopes could differ. Of course, if genes with pleiotropic effects on two traits were fixed within a population, there would be no correlated response to selection.

If genetic correlations are driven by linkage, we expect that populations with different frequencies of sex will differ in their correlated responses. However, specific predictions depend on the underlying distribution of allelic variants. We can consider a simplification of the problem where two traits, *A* and *B*, are governed by variation at two linked loci, *a* and *b*. In one form of allelic variation, alternate alleles at loci *a* and *b* lead to different slopes of association between traits *A* and *B*. If allelic variation takes this form, we expect there to be no net trait correlation within a population, and thus our clonal selection on body size would yield no response in a second trait.

Alternatively, alternate alleles at loci *a* and *b* may lead to trait variation that slides along a common slope. Such variation would lead to the observation of genetic correlations among traits within a population. In this case, correlations that arise out of chance linkage are expected to be quickly eroded away by recombination in populations with relatively high frequencies of recombination. Therefore, correlated responses to selection would be stronger in populations with lower frequencies of sex. As these associations arise out of chance events, there is no force driving correlations to be similar in different populations, and therefore we expect slopes of association to differ among populations.

The situation under correlations which arise from linkage due to selection is more complicated. As with chance linkage, any correlations which do arise are going to be eroded by recombination, and we might expect this to result in populations with high frequencies of recombination to have smaller genetic correlations between traits than in populations with lower recombination. However, this expectation ignores the role of recombination in novel allele combinations with high fitness, and it is possible that the creative potential of sex outweighs the erosive effects of sex. If the net effect of sex is beneficial, and a particular correlation holds a selective advantage, sex would be creating high fitness clones that adhere closely to the optimal trait correlation. In taxa with the potential for asexual reproduction, such as *Daphnia*, these fit clones can spread through demographic expansion, shielding highly favored combinations from sexual erosion.

If the erosion of favorable combinations outweighs their origin, the net effect of sex is detrimental. This may occur, for example, if the capacity for asexual demographic expansion is limited or if few of the potential combinations of alleles are in fact advantageous. In this case, the predicted differences between populations with high and low frequencies of sex are qualitatively similar to the situation under chance linkage: high-sex populations should have smaller correlated responses than low-sex populations. However, the magnitude of the difference would be smaller than in the chance linkage situation, because selection is still countering the erosion via sex to some degree.

## Methods

We divided our project into two phases. In the first phase, we isolated individual *D. pulicaria* from the field, established them as clonal lineages in the lab, and selected clones with extreme body sizes. In the second phase, we assayed life history characteristics of the selected clones in a common garden life table experiment.

### Source populations and clonal isolation

We chose four lakes with well-known populations of *D. pulicaria* (Bristol, Little Long, Pine, and Warner) near the W. K. Kellogg Biological Station in southwest Michigan, (Table S1). These populations have been the subject of extensive ecological study (e.g., Tessier and Welser 1991; Tessier and Leibold 1992; Geedey et al. 1996; Dudycha 2001, 2004; Tessier and Woodruff 2002a,b; Tessier & Cáceres 2004; Cáceres and Tessier 2003, 2004) and we were able to choose populations that have different levels of investment in sexual reproduction (Cáceres and Tessier 2004; Tessier & Cáceres 2004). In a common garden study, Tessier & Cáceres (2004) found that 6% of the clones from Bristol and Little Long never invested in sexual reproduction, whereas 36% of Pine clones and 45% of Warner clones never invested in sex. In contrast, 67% of Bristol clones and 38% of Little Long clones invested in sex through both female and male function. The equivalent numbers for Pine and Warner were 28% and 22%, respectively. Similarly, in a 3-year study of field demography, they found that sex investment in Little Long and Bristol averaged anywhere from 2.2 to 50 times higher than in Warner and Pine, depending on the specific lakes being compared and whether male or female function was considered (Cáceres and Tessier 2004).

In early April 2002, we sampled each lake repeatedly with vertical hauls of an 80-*μ*m mesh Wisconsin net. This is the time period of the year when clonal diversity is expected to be highest, because new clones will have recently emerged from the sexually produced egg bank. A total of 600 individual females were isolated from each population into 50-mL centrifuge tubes with 40 mL of filtered (1 *μ*m) lakewater. These were initially kept at 10°C, at a 12:12 L:D photoperiod and fed a standard lab preparation of the green alga *Scenedesmus auratus*. Animals were allowed to reproduce asexually and thus form clonal lineages. In three populations, ∼90% of the animals isolated resulted in lineages that were available at the start of the selection procedure. However, only about 50% of the animals isolated from Warner Lake yielded lineages. This resulted from a combination of relatively poor survivorship of the originally isolated individuals and a relatively high tendency for those who did survive to produce solely male offspring. In previous work on the Warner population (Dudycha and Tessier 1999), there was no indication that clonal lineages had lower establishment rates than other *D. pulicaria* populations.

A recent population genetic analysis (Allen and Lynch 2012) of a subset of clones (*n* = 60 per population) from our base population samples showed that observed heterozygosities in Bristol and Little Long matched expected heterozygosities, but that there was heterozygote excess in Pine and Warner. Inbreeding coefficient (F_IS_) estimates were 0.02 in both Bristol and Little Long, but significantly negative in Warner and Pine (−0.2 and −0.3, respectively). The ratio of observed to expected multilocus genotypic diversity was significantly lower than expected in Warner (ratio = 0.4), Pine (0.3), and Bristol (0.5), but not Little Long (1.0). These results are consistent with greater effects of clonal selection in populations with lower frequencies of sex.

### Selection protocol

We designed our selection protocol to identify the 10 largest and smallest clones from each population, with body size defined as the average length at maturity (from base of the tailspine to top of the head; maturity was defined as the first appearance of eggs in the brood chamber). Prior to the initiation of selection, clones were acclimated to the eventual life history assay conditions: 20°C, 12:12 L:D, and 3 × 10^5^ cells/mL of *Scenedesmus* fed daily. Experimental animals were transferred to new 250-mL beakers with 150 mL of filtered lakewater every other day. All beakers were kept in the same walk-in environmental chamber in randomized positions that were rotated haphazardly with each lakewater change. These conditions were maintained throughout the selection period and life history assay.

We selected on body size in two episodes. In the first, we identified approximately 30 large and small clones in each population. In the second, these were further reduced to the 10 largest and smallest. As it was impossible to perform the initial body size assay on all clones concurrently, we divided the first episode of selection into two blocks, quantifying body size on half of the clones from each population during each block.

To assay body size, we first divided each clonal lineage into two sub-lines in order to allow for any remaining maternal effects variation to be incorporated in within-clone variation (Lynch et al. 1989). Then, a single individual was haphazardly chosen from the 2nd clutch of each mother (i.e., two replicate individuals per clone, each from a different mother) and allowed to grow to maturity under the conditions described above. Starting when animals were 4 days old, they were examined daily for maturity, and when the first clutch of eggs appeared, they were measured with an ocular micrometer in a dissecting microscope at 50X. After adjusting for block effects in each population separately, we identified the ∼30 largest and smallest clones in each population. A few clones that would otherwise have been chosen were extinct by the start of the second body size assay, and these were replaced with the next biggest or smallest clones. Mothers of some clones produced male offspring during the experimental set-up, particularly in the Warner population, and as a result the number of clones on which we obtained body size data was fewer than our target of 400.

We conducted the second body size assay concurrently on all 234 clones selected in the first episode. In this assay, we followed a similar procedure to the first assay, except that each clonal lineage was divided into four maternal sub-lines, and we sought to measure two individuals in each sub-line, for a total of eight individuals per clone. Again, this was limited by male production by mothers and juvenile mortality, but for most clones, we had at least five observations of size at maturity. From these data, we identified the 10 largest and smallest clones in each population for use in our life history assay.

Size distributions of each population (Figure S1) and details on the clones selected (Table S3) are given in the Supplemental Information.

### Life history assay

After selecting for extreme body size in our four populations, we performed a life table experiment on the resultant 79 clones. Prior to the life table, we again allowed two generations to pass under standard conditions to minimize maternal variation. Then, each clone was divided into 10 replicate sub-lines, and a single female offspring from the 2nd clutch (or 3rd, if the 2nd was male) was chosen for the life table. These individuals were raised under the conditions described above, and observed daily. For each of the 10 replicate individuals per clonal lineage, we recorded length at birth, at maturity, and when the 4th clutch was released. We also recorded clutch size, offspring sex ratio, and date for the 1st through 4th clutches produced. From these data, we calculated composite traits including juvenile and adult specific growth rates (i.e., mass per mass per time), inter-clutch intervals and *r*, the intrinsic rate of increase.

### Data analysis and archiving

To assess the effectiveness of our selection protocol, we calculated selection differentials (*S*) by subtracting the overall mean body size of a group in a particular body size assay from the mean body size of the clones selected at the completion of that same assay. The response to selection (*R*) was calculated by subtracting the overall mean body size of a group from the mean of the selected clones as measured in the subsequent assay. Broad-sense heritability (*H*^2^) was then calculated as the ratio *R/S* (Lynch and Walsh 1998).

We tested for correlated responses to selection on size at maturity in each population separately with clone as our unit of replication. We used univariate two-tailed *t*-tests, correcting for multiple comparisons within populations via the Bonferroni method. We chose a univariate approach because we did not want to assume that all correlations were driven by the same mechanisms. When appropriate, variances were pooled across large- and small body size-selected clones, otherwise *t*-tests were based on separate variances. To test the specific hypothesis that frequency of sex influences the response to clonal selection, we constructed linear mixed models for each trait where at least one population showed a marginally significant difference between the large and small selected clones. These models used type III sums of squares, and included direction of selection (large or small), frequency of sex in the field (high or low), population nested within sex frequency (treated as a random effects variable), and an interaction between sex frequency and selection. The interaction provides the critical test of our core predictions.

We were interested in whether the slope of the association between size at maturity and other traits was similar across populations. We estimated regression slopes, and then tested whether they were significantly different among populations in separate linear models for each trait. In these models, the correlated trait was the dependent variable, with size at maturity, population, and a population-by-size at maturity interaction as independent variables. To determine if slopes are different across populations, we tested the significance of the interaction term.

All statistical analyses were conducted in Systat 12.0 (Systat Software, Inc., San Jose, CA). Archived phenotypic data can be accessed from Dryad under doi: 10.5061/dryad.fv3cb.

## Results

### Direct response to selection

Body size of clones was distributed approximately normally in each base population (Figure S1). As variances of clonal means were similar across populations (Table 1), there was no indication that high-sex populations (Bristol, Little Long) harbored more clonal variation for body size than the low-sex populations (Pine, Warner).

**Table 1:** Size at maturity in the base population of *Daphnia pulicaria* as measured in the first body size assay

Population	*N*	Mean size at maturity (mm)	S.D.
Bristol	362	1.680	0.205
Little long	367	1.725	0.183
Pine	395	1.474	0.187
Warner	184	1.774	0.183

*N* is the number of clones measured in a population, Mean Size at Maturity gives the body length from the base of the tailspine to the top of the head when eggs first appear in the brood chamber (averaged across clones after clonal estimates were adjusted for block effects), S.D. gives the standard deviation.

**Figure 2:**
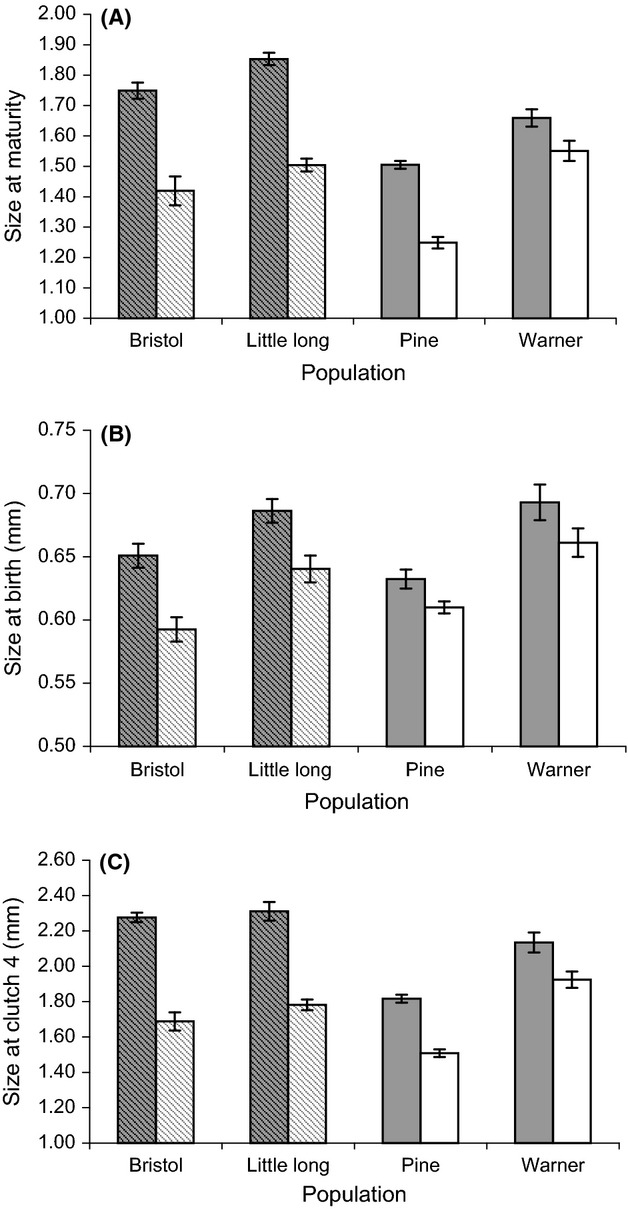
Divergence of body size between large (gray bars) and small (white bars) selected clones. Error bars show standard errors. Populations with relatively high frequencies of sex are indicated by diagonal lines across the bars. A) Direct response in size at maturity. B) Correlated response in size at birth. C) Correlated response in size when the 4^th^ clutch is released.

Approximately 30 clones in each size direction were chosen for a second assay of body size. This meant that we selected the upper and lower ∼10% of the size distribution, and produced selection differentials of approximately ±0.3, depending on the direction of selection (Table 2). Responses to selection in this episode were substantial, but asymmetrical, and the corresponding realized broad-sense heritabilities ranged from 0.30 to 0.85 (Table 2). Based on the second body size assay, we further reduced the number of clones to the 10 largest and smallest clones in each population (2.5–5.4% of the base population). This generally did not lead to increased differentiation within populations (Table S2), suggesting that the first episode of selection captured most of the variability present in the base population. Selection differentials for the second episode of selection (0.08–0.09) were approximately one-fourth the magnitude of selection differentials in the first round, and six of eight responses to selection in the second episode were in the opposite direction to the selection applied (Table S2). However, in all four populations, the combined selection yielded absolute differences in the mean size at maturity between final large and small clones during the life table assay (Fig. 2; *P* < 0.0001 in all cases except Warner, where *P* = 0.0229). Size differences of the final set of clones selected from the second body size assay ranged from 0.45 mm (Warner) to 0.68 mm (Little Long). This meant that the average size of large clones was 30% larger than small clones in the Warner population, 39% larger in Little Long, 51% larger in Pine, and 53% larger in Bristol (Table S2).

**Table 2:** Body size and its response to selection in the first episode of selection

Population	Direction	*N*	Mean 1	*S*	Mean 2	*R*	*H*^2^
*High sex*							
Bristol	Large	30	2.013	0.333	1.864	0.185	0.55
	Small	29	1.317	−0.363	1.397	−0.283	0.78
Little long	Large	32	2.048	0.323	1.902	0.177	0.55
	Small	32	1.400	−0.325	1.560	−0.165	0.51
*Low sex*							
Pine	Large	30	1.833	0.359	1.690	0.216	0.60
	Small	29	1.166	−0.308	1.240	−0.234	0.76
Warner	Large	29	2.071	0.298	1.862	0.088	0.30
	Small	23	1.550	−0.224	1.583	−0.190	0.85

*N* is the number of clones that were selected from the base population and measured in the second body size assay. Mean 1 and Mean 2 are the mean body sizes (in mm) of these clones in the first and second body size assays, respectively. *S* is the selection differential, *R* is the response to selection, and *H*^2^ is the broad-sense heritability.

No association was evident between realized broad-sense heritability (*H*^2^) and sex frequency or mean body size for the four populations. However, in three of the four populations, *H*^2^ was higher when selecting for small body size (Table 2).

### Correlated responses to selection

In general, size and reproductive output traits diverged between the large and small lines in all populations, whereas time-based traits did not diverge consistently (Table 3). For some traits, notably clutch sizes, adult specific growth rate, and L_4_, the magnitude of correlated responses were higher in populations that have high-sex investment in the field (Bristol, Little Long) than those with low-sex investment in the field (Pine, Warner). Linear models showed a significant sex frequency-by-selection interaction in L_4_, reproductive output, and adult specific growth rate, and marginally significant interactions for L_0_ and *r* (Table 4). In all of these interactions, the correlated response was higher in the high-sex populations than in the low-sex populations. In addition, more traits were significantly different in the high-sex populations than in the low-sex populations (see Table S4 for details).

**Table 3:** Mean values of life history traits for the large- and small body size-selected groups in four populations of *Daphnia pulicaria*

Population	Selection direction	L_M_	L_0_	L_4_	C_1_	C_2_	C_3_	C_4_	C_1-4_
Bristol (High sex)	Large	1.75	0.65	2.28	6.09	12.30	14.15	15.65	48.85
		0.03	0.01	0.03	0.54	0.93	0.68	0.89	2.61
	Small	1.42	0.59	1.69	3.02	5.41	5.54	5.80	19.67
		0.05	0.01	0.05	0.43	0.74	0.71	0.81	2.57
Little long (High sex)	Large	1.85	0.69	2.31	6.43	13.86	15.17	15.68	51.50
		0.02	0.01	0.05	0.35	0.78	0.97	0.73	2.64
	Small	1.50	0.64	1.78	3.94	6.96	7.67	7.39	26.09
		0.02	0.01	0.03	0.30	0.32	0.49	0.43	1.32
Pine (Low sex)	Large	1.50	0.63	1.82	4.84	7.61	7.64	7.85	27.82
		0.01	0.01	0.02	0.30	0.27	0.55	0.42	1.02
	Small	1.25	0.61	1.51	3.50	4.45	4.41	4.59	16.83
		0.02	0.00	0.02	0.17	0.46	0.35	0.40	1.29
Warner (Low sex)	Large	1.66	0.69	2.13	5.10	11.06	11.55	11.93	39.99
		0.03	0.01	0.06	0.57	1.22	0.91	0.74	2.70
	Small	1.55	0.66	1.92	4.69	7.87	7.72	7.84	28.13
		0.03	0.01	0.05	0.22	0.41	0.30	0.54	1.32

Population	Selection direction	Age at maturity	g_Juve_	g_Ad_	ICI	Sex ratio	*r*
Bristol (High sex)	Large	8.54	0.170	0.025	2.48	0.03	0.613
		0.12	0.004	0.002	0.04	0.02	0.015
	Small	8.02	0.169	0.017	2.46	0.00	0.521
		0.12	0.012	0.001	0.05	0.00	0.025
Little long (High sex)	Large	8.13	0.166	0.022	2.50	0.00	0.661
		0.15	0.005	0.002	0.06	0.00	0.018
	Small	8.11	0.167	0.016	2.48	0.00	0.580
		0.18	0.005	0.001	0.05	0.00	0.009
Pine (Low sex)	Large	8.20	0.161	0.019	2.42	0.00	0.604
		0.14	0.007	0.001	0.03	0.00	0.013
	Small	7.92	0.147	0.017	2.59	0.00	0.517
		0.11	0.008	0.002	0.06	0.00	0.016
Warner (Low sex)	Large	8.45	0.160	0.023	2.51	0.02	0.604
		0.22	0.004	0.001	0.06	0.01	0.024
	Small	7.89	0.157	0.020	2.57	0.02	0.603
		0.13	0.003	0.001	0.04	0.02	0.013

*N* = 10 clones for all traits in all groups, except Bristol Low, where *N* = 9. Standard errors are reported below means. L_0_, L_M_, and L_4_ are the body lengths (in mm) at birth, at maturity, and when the 4th clutch was released, respectively. C_1_, C_2_, C_3_, and C_4_ are the number of offspring in the first through fourth clutches, C_1–4_ is the sum across these clutches. Age at maturity is the age (in days) when eggs first appeared in the brood chamber. g_Juve_ and g_Ad_ are juvenile and adult specific growth rates (mass per mass per day). ICI is the inter-clutch interval, averaged over clutches 1–4. Sex ratio is the offspring sex ratio across clutches 1–4. *r* is the intrinsic population growth rate, estimated from age-specific survival and reproduction.

**Table 4:** Interactions between clonal selection and historical frequency of sex as estimated in a linear model with sex frequency, population nested within sex frequency (treated as a random effects variable), direction of selection, and a sex frequency-by-direction of selection interaction as factors. For all interaction tests, df = 1, 73

Trait	F-ratio	*P*
Length at 4th clutch	26.9857	<0.0001
Length at birth	3.1672	0.0793
Total offspring (Clutches 1–4)	30.3766	<0.0001
Adult specific growth rate	4.9726	0.0288
Age at maturity	0.4718	0.4941
*r*	2.7668	0.1005

We measured the correlated response to selection in two size traits: body length at birth (L_0_) and again when the fourth clutch (L_4_) was released (Fig. 2). Unsurprisingly, both responded to clonal selection on size at maturity in all four populations, although the differences in L_0_ were not statistically significant in the low-sex populations and in Warner the L_4_ difference was only marginally significant. On a percentage basis, the responses of L_4_ were higher than L_0_ in all populations (Table S5). L_0_ is not expected to be as tightly associated with size at maturity as L_4_ because it is the product of tradeoffs between offspring size and number, in addition to tradeoffs among survival, growth, and total reproductive investment. Response of L_0_ suggests that low-sex populations have a smaller correlated response than high-sex populations largely because the response of L_0_ in Pine Lake was notably small, with the large-selected Pine neonates only 3.7% longer than small-selected Pine neonates. In the high-sex populations, the comparable differences were 9.9% (Bristol) and 7.2% (Little Long) (Fig. 2; Table 4).

**Figure 3:**
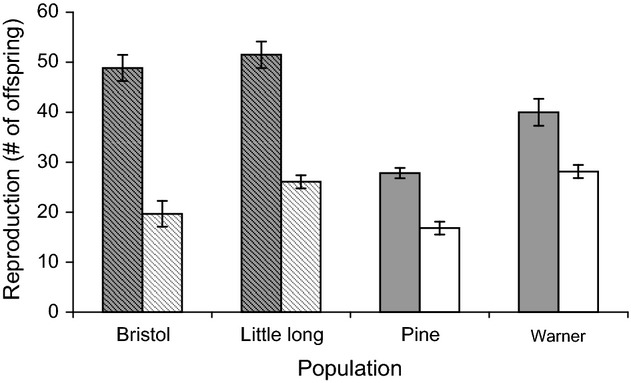
Correlated divergence of reproduction in clones selected for large (gray bars) or small (white bars) body size. Error bars show standard errors. Populations with relatively high frequencies of sex are indicated by diagonal lines across the bars. Reproduction includes all offspring produced in the first four clutches.

Correlated responses of the number of offspring produced were stronger in high-sex populations than in low-sex populations (Fig. 3), but were statistically significant in all populations. Patterns were similar across all four clutches in which offspring were counted (Figure S2), so we show here only the reproductive output summed across all clutches.

**Figure 4:**
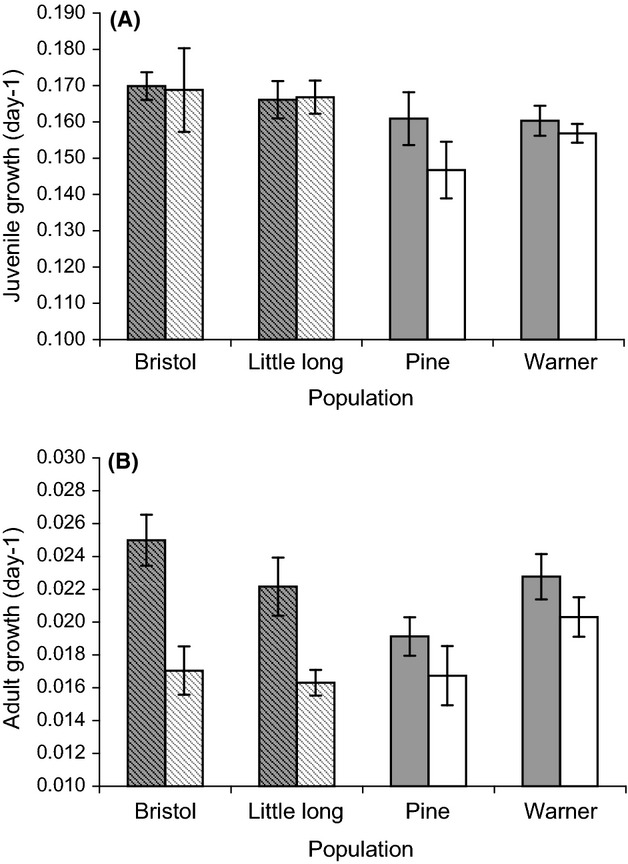
Correlated divergence of growth in clones selected for large (gray bars) or small (white bars) body size. Error bars show standard errors. Populations with relatively high frequencies of sex are indicated by diagonal lines across the bars. A) Adult specific growth rate between maturity and release of the fourth clutch. B) Juvenile specific growth rate from birth to maturity.

Growth rates integrate elements of both body size and time, and thus correlations with size at a particular developmental point may be weak. Differences in adult specific growth rate were seen in all populations, but were statistically significant in only the high-sex populations (Fig. 4A). Note that adult specific growth is not simply a recapitulation of L_4_ because it is scaled to body size. In contrast, juvenile specific growth rate showed no correlated response to selection on size at maturity in three of the four populations (Fig. 4B), and the difference was not statistically significant in the fourth. Interestingly, the potential for a correlated response occurred in Pine Lake, which was also the population that showed the diminished response in size at birth. This suggests that size at birth and juvenile growth play different roles in determining adult size variation in different populations.

**Figure 5:**
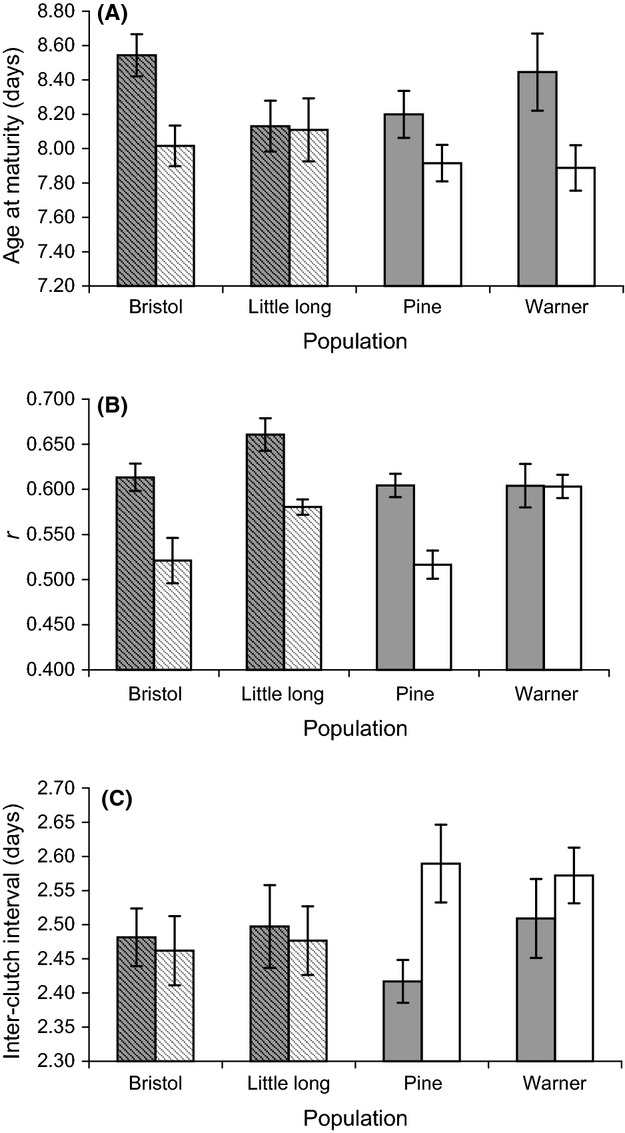
Correlated divergence of traits in clones selected for large (gray bars) or small (white bars) body size. Error bars show standard errors. Populations with relatively high frequencies of sex are indicated by diagonal lines across the bars. A) Age at maturity. B) Intrinsic rate of increase. C) Inter-clutch interval.

Age at maturity integrates elements of both time and reproduction, but does not directly incorporate body size. It appeared to diverge in three of the four populations (Fig. 5A), but there was no association with frequency of sex or magnitude of the direct selection response. Bristol and Warner showed the highest correlated response of age at maturity, Pine showed a modest response, and Little Long showed none. Except for marginal significance in Bristol (Table S4), these apparent differences were not statistically significant after correction for multiple comparisons.

We estimated *r*, the intrinsic rate of increase, following McGraw and Caswell (1996). Like age at maturity, it depends on both timing and reproduction, and does not directly incorporate body size. Response of *r* to clonal selection on body size was quite substantial, with large-body size clones having an advantage of ∼0.1 over small-body size clones, in three of the four populations (Fig. 5B). These differences were significant for Pine and Little Long, and marginally so for Bristol. In Warner, however, there was no divergence of *r*. Although the correlated responses may seem to imply large-bodied clones should displace small-bodied clones, the lab environment protects individuals from size-selective mortality, and thus our estimates of *r* should be interpreted as an index of physiological capacity, not fitness in the field.

Finally, we assayed the response of the inter-clutch interval (ICI). ICI is not strictly a reproductive feature in *Daphnia*, as it is tied to the molt cycle, with molting, growth, and release of offspring all synchronized. ICI was the only trait where the correlated response appeared greater in the low-sex populations than the high-sex populations, but none of these differences were statistically significant (Fig. 5C).

### Comparison of regression slopes

Regression analysis unsurprisingly paralleled correlated divergence, with significant regression slopes generally for size, reproduction, and *r*. Age at maturity, adult growth, and inter-clutch interval each had a significant association in one population (Bristol, Little Long, and Pine, respectively). Linear models testing whether slopes differed across populations (i.e., an interaction between population and size at maturity) found no support for differences except in size at birth (*F*_3,71_= 3.5455, *P* = 0.0187). Detailed results from the regression analysis are given in Supplementary Tables 5 and 6.

## Discussion

Understanding phenotypic evolution for a trait requires an understanding of the indirect selection pressures it experiences via genetic correlations. Those indirect pressures are a consequence of both the selection exerted on correlated traits, and the mechanisms of correlation by which phenotypic traits can be connected. We have experimentally evaluated the correlated responses to clonal selection on body size in populations that differ in their history of sexual recombination with the aim of understanding the mechanisms of connection. These differences allow us to potentially distinguish among pleiotropy, chance linkage, and selective advantage as mechanisms underlying genetic correlations. Furthermore, sexual recombination itself remains a significant puzzle in evolutionary biology because it simultaneously has the capacity to demolish and assemble genomes with high fitness (Lehtonen et al. 2012; Meirmans et al. 2012); this puzzle is partly about the effects of recombination on genetic correlations. Our results show that the magnitude of correlated responses to selection for some critical life history traits is higher in populations whose genetic composition is the result of frequent sex, despite the observation that the slope of the associations between life history traits and body size do not differ among populations according to historical frequency of sex. Together, these results provide a novel line of support for the hypotheses that selective processes are substantial contributors to life history correlations and that sexual recombination promotes adaptation by natural selection.

### Mechanisms of correlation

We observed significant correlated responses to selection on body size at maturity in one or more populations for most traits (specifics given in Tables 3 and S4). Although details for particular traits differ, these generally confirm the observation that life history traits are associated with size at maturity.

For three traits (body size at the release of the fourth clutch, total reproduction, and adult specific growth rate), the magnitude of divergence was clearly greater in populations with a high historical frequency of sex, and a similar trend was evident in two other traits (size at birth and *r*). Of the potential mechanisms contributing to trait associations, this was predicted only when there was both selection for a particular correlation and the net effect of the higher frequency of sex was to promote adaptation through the creation of high fitness clones. If this is true, we would predict that the strengths of the correlations are tighter in the high-sex populations than in the low-sex populations. However, we cannot evaluate this prediction effectively because we did not conduct the final life table assay on the full set of clones initially isolated. Although our clonal selection procedure allows us to estimate the slope of the relationship efficiently, the absence of the middle-value clones from the life table means that we do not know how tight that relationship is.

Only the inter-clutch interval (equivalent to instar duration) suggested greater divergence in low-sex populations. This could be consistent with a role for chance linkage or selection, if the net effect of sex was deleterious. There was no statistical support for differences among populations in the slope of the relationship (Table S5), which is consistent with the selection in the context of deleterious sex. However, none of the observed divergences were statistically significant, so it is unreasonable to lend much weight to this interpretation.

We found little support for the notion that the association between body size and other life history traits differed among populations, except for size at birth. In principle, this can arise under any of the mechanisms contributing to genetic correlations. There was no significant difference between high and low-sex populations, but the trend was for high-sex populations to have a larger difference than the low-sex populations. This is opposite of what is expected under chance linkage. Under the mechanism of pleiotropies arising from different genes in different populations, there should be no effect of sex. However, even though the trend is insignificant, it is worth considering the possibility that selection is contributing to the correlation between size at maturity and size at birth, but that the optimal correlation differs among populations (discussed below).

If genetic correlations among life history traits were largely driven by pleiotropies, recombination frequency should have no effect on the divergence of correlated traits when selection is applied directly to a key trait. In this case, we would expect to see the same degree of correlated divergence in all populations if each population harbored the same distribution of allelic variation, but this occurred for none of the traits examined. The magnitude of correlated divergence in age at maturity was idiosyncratic with respect to sex, with no evidence that the association slope differed among populations. This is consistent with pleiotropies that emerge from the same genes in each population, if allelic variation differs among populations.

Given that the traits we measured differ in their similarity to size at maturity, the directly selected trait, we expected some traits, particularly body size at later adulthood, to be driven by pleiotropy. For example, it seems reasonable that the shared genetic basis of size at other ages would be strong, of reproductive output (which in *Daphnia* is constrained by brood chamber size) would be moderate, and of timing traits would be small. Instead, traits from all three classes showed a strong effect of historical frequency of sex. This should not be interpreted to mean that pleiotropy is absent from these correlations, as it is possible for there to be a shared pleiotropic basis for a correlation that is common to all populations onto which selection builds the correlation further.

Collectively, our results show that the relative contribution of alternative mechanisms of correlation may differ among traits, but that selection is a key driver of life history correlations with size at maturity in *Daphnia pulicaria*. Our data should not be interpreted to mean that selective correlations are solely responsible for trait correlations. Rather, the complexity of our results shows that no single mechanism behind genetic correlations among complex traits provides satisfactory explanations for variation of the traits. Linkage and pleiotropies are likely to also contribute to the variation, and research aimed at identifying specific pathways that drive extant life history variation, coupled with studies that describe the physical relationships of key genes within genomes in the context of recombination frequency will be necessary.

In comparing our study to others that have sought to distinguish among correlation mechanisms, we do not see common patterns emerging, but the relevant studies have been few. Conner (2002) conducted an experiment in which he studied floral traits in large experimental populations of wild radish. He concluded that correlations among six floral traits were pleiotropic, because nine generations of enforced random mating failed to diminish the strength of genetic correlations. In an analysis of QTLs associated with the response to selection on bristle number in *Drosophila*, Nuzhdin et al. (1999) concluded that responses in correlated bristle traits were likely due to linkage, rather than pleiotropy, but did not attempt to distinguish between adaptive and chance correlations. Delph et al. (2011) investigated the basis of the genetic correlation between male and female flower size in *Silene latifolia* in a selection experiment where they sought to break the correlation. Despite prior evidence suggesting a role for pleiotropy (Delph et al. 2004), in one selection line they were able to eliminate the genetic correlation almost entirely in a few generations of selection, and concluded that selection was likely to be the fundamental cause of the correlation in nature.

We note that these other studies have focused on morphological traits rather than life history. Before generalizations can emerge, further analyses of this sort are necessary, particularly in organisms such as *Raphanus*, *Silene*, and *Daphnia* where the ecological consequences of trait variation can be evaluated.

### Selection on correlations

We have argued that our four populations are ecologically similar, and in the broader context of potential habitats, they are. From the perspective of zooplankton like *Daphnia*, the most significant ecological differences occur along a gradient of waterbody size (Wellborn et al. 1996; Tessier and Woodruff 2002a) and a gradient of productivity. These differences include the shift from temporary to permanent waters, the shift to where a lake can support planktivorous fish and, at even larger sizes, piscivorous fish, and finally fully stratified lakes with a hypolimnion. It is not clear whether *Daphnia pulicaria* is a recently derived species or an ecologically defined variant of *D. pulex* and thus it is hard to define the niche breadth of *D. pulicaria*. There is ample evidence of natural hybridization (reviewed in Heier and Dudycha 2009), gene flow across the major habitat transitions (Dudycha 2004; Crease et al. 2011; Cristescu et al. 2012), and the absence of reproductive isolation in experimental crosses between stratified lake populations and temporary pond populations (Heier and Dudycha 2009). All four of the populations in this study are stratified lakes with planktivores (mainly bluegill) and piscivores (smallmouth bass), and other species of *Daphnia* (mainly *D. dentifera*) as a primary competitor. In addition, all four lakes have similar productivity, near the upper part of what is considered oligotrophic, as estimated from Spring measures of total phosphorus (Cáceres and Tessier 2004).

Nonetheless, the lakes are not identical, and this creates the potential for selection for differences in the precise correlations among traits. The predictions we described with regard to similarity or dissimilarity of correlations across populations are based on the assumption that the same correlations are selected for in each population. For the traits where we have inferred an important role for selection due to greater correlated differences in the higher sex populations, it is hard to see what could lead to similar correlations across populations if there were in fact selection for different correlations. Even if there were real differences in slope that we did not have the power to detect, that would still implicate selection as the driving mechanisms behind the correlations. In fact, the slope of the association with size at birth was different across populations, and this may be due to different selection pressures.

We have detected a quantitative genetic signature of selection on some correlations, but we do not have data that test the fitness consequences of the correlations in the wild. Although we estimated *r*, this is not indicative of fitness of *Daphnia* in lakes, where seasonal succession and other environmental variation may have strong impacts on what traits and trait combinations are advantageous at different times. Long-term fitness in *Daphnia* in the field will depend on the contribution of an individual to future generations via both asexual and sexual reproduction, and genotypes may differ in the reproductive mode in which they have an advantage. It would be very interesting to compare the field performance of clones that differ in body size and degree of deviation from the correlation in their population.

### The creative power of sex

Although genetic correlations of life history traits and the evolutionary consequences of recombination have been studied extensively, few attempts have been made to juxtapose them in a single study. This is somewhat surprising, because an essential element of debate about the evolutionary role of recombination is the direction and extent to which it influences favorable combinations of traits. Relatively few study systems offer an opportunity to consider populations that vary in recombination frequency, and most considerations of the effect recombination has on correlations have focused on examining genetic correlations before and after a limited number of rounds of recombination in exclusively sexual species. In our system, the only situation that is expected to yield stronger correlated responses to selection in high-sex populations compared with low sex is when genetic correlations result from selection for the correlation and the creative power of sex outweighs its destructive power. For *Daphnia*, capitalizing upon the power of sex to create advantageous combinations of alleles probably depends on the opportunity for fit clones to expand through asexual reproduction. This raises the question of what constraints operate to determine an optimal frequency of sex in cyclic parthenogens, as it is easy to imagine that as the frequency of sex increases, the expansion of fit clones would decrease.

## Conclusion

It is apparent that no single approach will provide a general understanding of the evolution of complex traits. Currently, there is a creative tension between molecular methods that seek to reduce complex traits to variation at the nucleotide level, and quantitative genetic methods that seek to integrate the multifaceted influences on complex traits. Both are important avenues of research, as we have yet to reach a point where general, predictive principles can accurately define future evolutionary trajectories of interlinked, complex traits. The recent sequence of the *Daphnia* genome, and the long history of demographic and trait-based ecological work, means that *Daphnia* provide the opportunity to combine approaches with studies like ours to gain a more complete picture of complex trait evolution.

## Supporting Information

**Figure S1.** Size distribution of clones in the base populations as measured in the first body size assay. Bars are labeled with the floor of each size class, in mm.

**Figure S2.** Correlated divergence of clutch size in the first through fourth clutch in the clones selected for large (gray bars) or small (white bars) body size. Error bars show standard errors. Note that vertical axes have different scales.

**Table S1.** Lake Locations.

**Table S2.** Body size and its response to selection in the second episode of selection. *N* is the number of clones that were selected from the second body size assay and measured in the life table assay. Mean 2 and Mean LT are the mean body sizes (in mm) of these clones in the second body size assay and life table assay, respectively. *S* is the selection differential, *R* is the response to selection, and *H*2 is the broad-sense heritability.

**Table S3.** Clones selected in the first body size assay and then assayed again in the second body size assay.

**Table S4.** Statistical tests of correlated divergence between large and small selected lines. Variance indicates whether variances were pooled or kept separate between selection and direction, df shows the degrees of freedom, *t* gives the test statistic. Traits are as follows: L4 = length when the 4th clutch was released, L0 = length at birth, C1–4 = total offspring in the first four clutches, g_Adult = adult specific growth rate, g_Juve = juvenile specific growth rate, *r* = intrinsic rate of increase, A = age at maturity, ICI = inter-clutch interval.

**Table S5.** Regression analysis showing relationship between size at maturity and other life history traits. Slope indicates the regression coefficient; S.E. gives the standard error of the coefficient. Significance tests are taken, all have df = 1, 18 (df = 1, 17 for traits in Bristol) and are Bonferroni corrected for multiple comparisons within populations.

**Table S6.** Tests of whether regression slopes in Table S5 differ among populations (i.e., whether a significant interaction between population and size at maturity is evident). For all traits, df = 3, 71 for the interaction term.
