# Supplementary material for: Correlated responses to clonal selection in populations of Daphnia pulicaria: mechanisms of genetic correlation and the creative power of sex
Source: Ecol Evol. 2012 Dec 19;3(2):204–16. doi: 10.1002/ece3.444 (PMC3586631; doi:10.1002/ece3.444)
Supplement: Supplementary file 1 [file ece30003-0204-SD1.pdf]

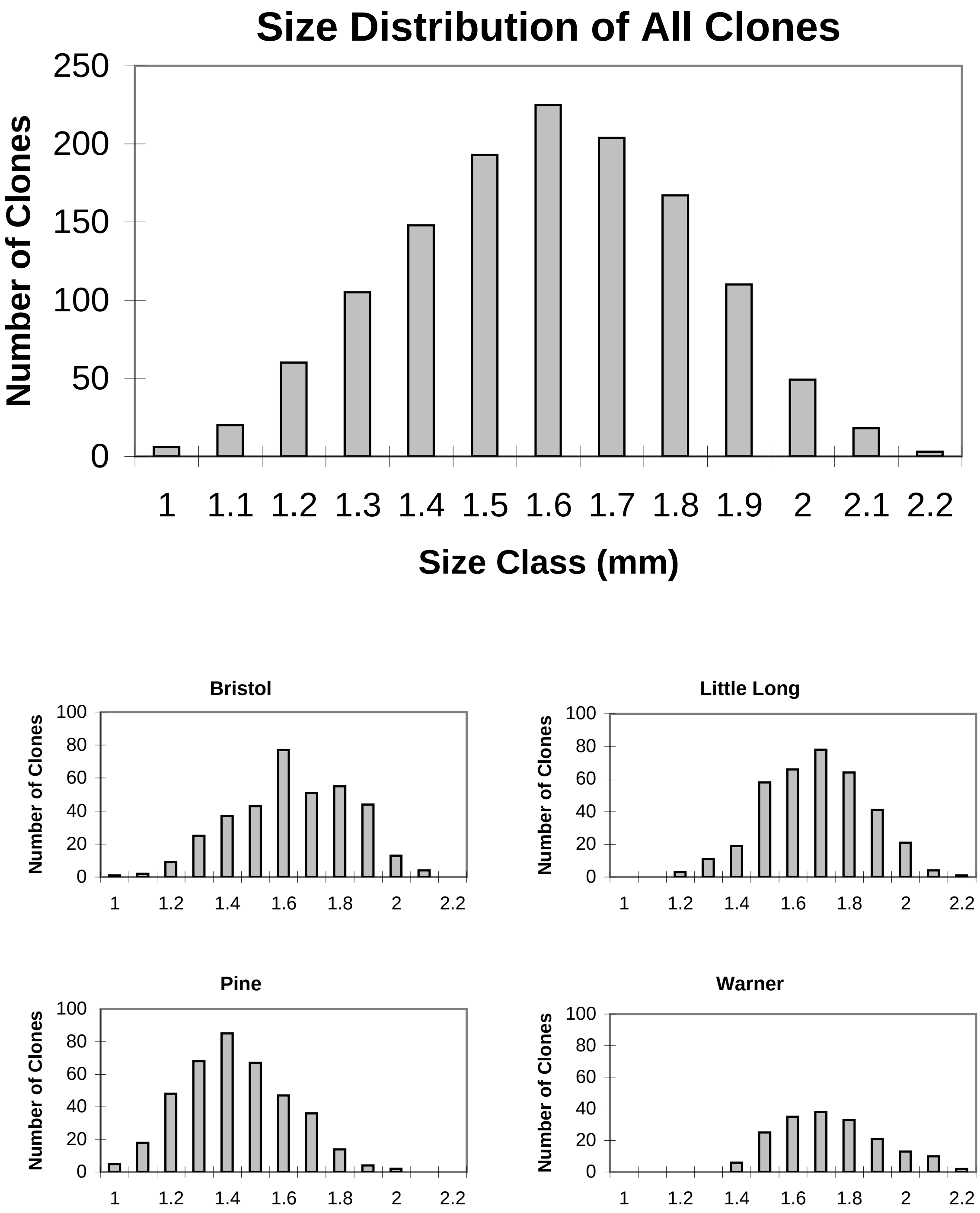

Figure S1: Size distribution of clones as measured in the first body size assay.
Bars are labeled with the floor of each size class, in mm.
