# Supplementary figures and images for: Correlated responses to clonal selection in populations of Daphnia pulicaria: mechanisms of genetic correlation and the creative power of sex
Source: Ecol Evol. 2012 Dec 19;3(2):204–16. doi: 10.1002/ece3.444 (PMC3586631; doi:10.1002/ece3.444)

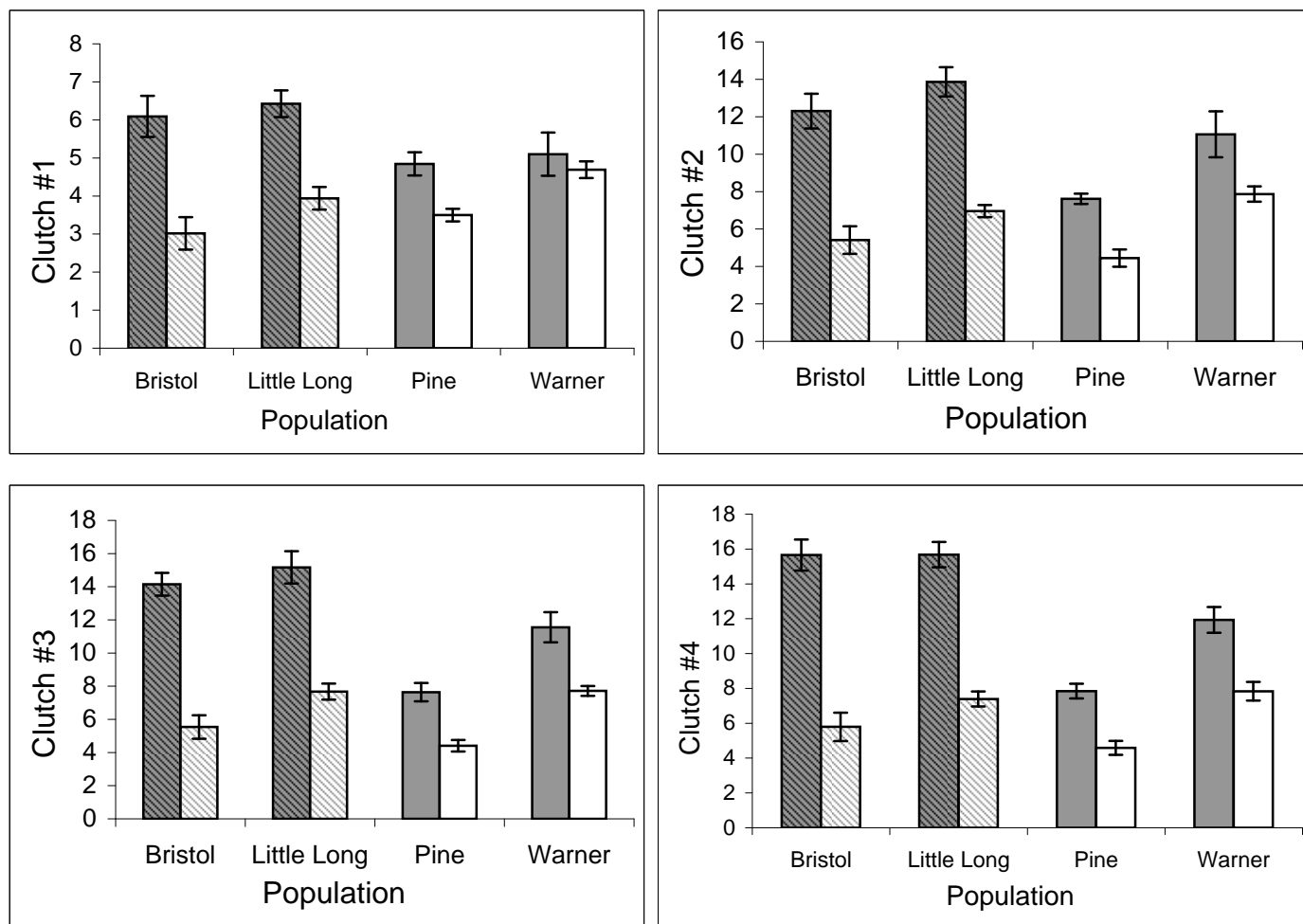

Figure S2

Supplement: Supplementary file 2 [file ece30003-0204-SD2.pdf]
