# Supplementary material for: Correlated responses to clonal selection in populations of Daphnia pulicaria: mechanisms of genetic correlation and the creative power of sex
Source: Ecol Evol. 2012 Dec 19;3(2):204–16. doi: 10.1002/ece3.444 (PMC3586631; doi:10.1002/ece3.444)
Supplement: Supplementary file 3 [file ece30003-0204-SD3.pdf]

Supplemental Information

Table S1. Lake Locations.

Lake	Latitude	Longitude
Bristol	42.4863° N	85.2481° W
Little Long	42.4220° N	85.4440° W
Pine	42.5738° N	85.3917° W
Warner	42.4694° N	85.5262° W

Table S2. Body size and its response to selection in the second episode of selection. *N* is the number of clones that were selected from the second body size assay and measured in the life table assay. Mean 2 and Mean LT are the mean body sizes (in mm) of these clones in the second body size assay and life table assay respectively. *S* is the selection differential, *R*is the response to selection, and H^2 is the broad-sense heritability.

Population	Direction	*N*	Mean 2	*S*	Mean LT	*R*	H^2
Bristol	Large	10	1.953	0.089	1.749	-0.115	
	Small	10	1.276	-0.121	1.455	0.058	
Little Long	Large	10	1.996	0.094	1.853	-0.048	
	Small	10	1.437	-0.125	1.504	-0.056	0.45
Pine	Large	10	1.765	0.075	1.505	-0.185	
	Small	10	1.171	-0.068	1.249	0.009	
Warner	Large	10	1.940	0.078	1.659	-0.202	
	Small	10	1.492	-0.091	1.551	-0.033	0.36

Table S3. Clones selected in the first body size assay and then assayed again in the second body size assay.

Selection Direction	Pop	Clone	Mean Length in 1st Assay	Mean Length in 2nd assay (mm)	Used in Life Table?
H	Bristol	7	2.04	1.844	
H	Bristol	9	2.12	1.949	yes
H	Bristol	15	1.98	1.847	
H	Bristol	32	1.96	1.889	
H	Bristol	35	1.98	1.924	yes
H	Bristol	72	2.06	1.92	
H	Bristol	89	1.96	1.822	
H	Bristol	90	2.02	1.871	
H	Bristol	108	1.98	1.938	yes
H	Bristol	161	1.98	1.794	
H	Bristol	169	2.05	1.957	yes
H	Bristol	176	2	1.798	
H	Bristol	187	2.05	1.953	yes
H	Bristol	204	2.1	1.936	yes
H	Bristol	207	2.03	2.037	yes
H	Bristol	345	2.01	1.946	yes
H	Bristol	351	2.13	1.784	
H	Bristol	360	2.01	1.856	
H	Bristol	366	1.99	1.64	
H	Bristol	384	1.99	1.962	yes
H	Bristol	390	1.97	1.932	yes

H	Bristol	429	2.19	1.843	
H	Bristol	463	2.01	1.786	
H	Bristol	478	2.01	1.804	
H	Bristol	481	1.97	1.793	
H	Bristol	490	2	1.794	
H	Bristol	495	2.03	1.829	
H	Bristol	513	1.99	1.715	
H	Bristol	538	1.97	1.922	
H	Bristol	543	1.99	1.848	
L	Bristol	16	1.08	1.156	yes
L	Bristol	54	1.36	1.416	
L	Bristol	61	1.34	1.486	
L	Bristol	71	1.36	1.29	yes
L	Bristol	106	1.36	1.569	
L	Bristol	109	1.21	1.346	yes
L	Bristol	152	1.28	1.337	yes
L	Bristol	153	1.16	1.498	
L	Bristol	159	1.34	1.559	
L	Bristol	202	1.26	1.376	
L	Bristol	234	1.36	1.531	
L	Bristol	239	1.34	1.397	
L	Bristol	249	1.34	1.54	
L	Bristol	301	1.29	1.618	
L	Bristol	304	1.34	1.336	
L	Bristol	311	1.33	1.12	yes
L	Bristol	314	1.27	1.288	yes
L	Bristol	321	1.1	1.424	
L	Bristol	329	1.33	1.399	

L	Bristol	335	1.35	1.364	yes
L	Bristol	363	1.27	1.463	
L	Bristol	411	1.33	1.411	
L	Bristol	413	1.35	1.26	yes
L	Bristol	425	1.29	1.439	
L	Bristol	436	1.29	1.382	
L	Bristol	453	1.33	1.408	
L	Bristol	460	1.29	1.261	yes
L	Bristol	527	1.33	1.386	
L	Bristol	532	1.33	1.448	
H	Litlong	20	2.15	1.995	yes
H	Litlong	23	2.01	1.82	
H	Litlong	69	2.16	1.962	yes
H	Litlong	80	2.05	1.778	
H	Litlong	82	2.13	1.82	
H	Litlong	97	2.07	1.904	
H	Litlong	101	2.05	1.908	
H	Litlong	121	2.01	1.56	
H	Litlong	141	2.04	1.843	
H	Litlong	161	2.05	1.917	
H	Litlong	167	2.05	1.981	yes
H	Litlong	215	2.09	1.809	
H	Litlong	244	1.99	2.005	yes
H	Litlong	301	2.04	1.843	
H	Litlong	322	1.98	1.963	yes
H	Litlong	327	2.05	1.973	yes
H	Litlong	330	2.05	1.886	
H	Litlong	334	1.98	1.987	yes

H	Litlong	339	2.24	2.009	yes
H	Litlong	351	2.07	2.078	yes
H	Litlong	364	2.06	1.953	
H	Litlong	365	1.97	1.842	
H	Litlong	386	2.04	1.84	
H	Litlong	387	1.99	1.781	
H	Litlong	404	1.97	1.878	
H	Litlong	451	1.98	1.881	
H	Litlong	454	2.02	1.922	
H	Litlong	464	1.98	1.907	
H	Litlong	470	2	1.941	
H	Litlong	487	2.03	1.923	
H	Litlong	529	2.16	1.951	
H	Litlong	534	2.06	2.005	yes
L	Litlong	27	1.32	1.46	yes
L	Litlong	99	1.44	1.563	
L	Litlong	104	1.37	1.425	yes
L	Litlong	123	1.44	1.437	yes
L	Litlong	127	1.5	1.566	
L	Litlong	179	1.5	1.704	
L	Litlong	189	1.45	1.558	
L	Litlong	193	1.47	1.593	
L	Litlong	238	1.38	1.738	
L	Litlong	310	1.36	1.602	
L	Litlong	333	1.36	1.57	yes
L	Litlong	337	1.3	1.363	yes
L	Litlong	348	1.47	1.728	
L	Litlong	357	1.49	1.689	

L	Litlong	360	1.44	1.504	
L	Litlong	379	1.38	1.626	
L	Litlong	418	1.48	1.675	
L	Litlong	431	1.44	1.432	yes
L	Litlong	439	1.48	1.517	
L	Litlong	467	1.44	1.483	yes
L	Litlong	469	1.22	1.546	
L	Litlong	471	1.3	1.416	yes
L	Litlong	473	1.32	1.626	
L	Litlong	483	1.42	1.637	
L	Litlong	486	1.48	1.506	
L	Litlong	491	1.48	1.703	
L	Litlong	495	1.32	1.603	
L	Litlong	501	1.2	1.492	
L	Litlong	523	1.25	1.375	yes
L	Litlong	524	1.43	1.473	yes
L	Litlong	538	1.44	1.658	
L	Litlong	550	1.43	1.664	
H	Pine	12	1.83	1.643	
H	Pine	27	1.91	1.677	
H	Pine	68	1.76	1.613	
H	Pine	76	1.82	1.668	
H	Pine	113	1.86	1.692	
H	Pine	117	1.9	1.713	
H	Pine	178	1.89	1.737	yes
H	Pine	186	1.83	1.568	
H	Pine	191	1.97	1.767	yes
H	Pine	220	1.87	1.435	

H	Pine	225	1.75	1.63	
H	Pine	234	1.76	1.783	yes
H	Pine	306	1.91	1.741	yes
H	Pine	321	1.73	1.695	
H	Pine	341	1.78	1.665	
H	Pine	358	1.81	1.71	
H	Pine	361	1.79	1.691	
H	Pine	364	1.77	1.61	
H	Pine	365	1.76	1.747	yes
H	Pine	381	1.84	1.656	
H	Pine	387	1.81	1.628	
H	Pine	390	2.04	1.77	yes
H	Pine	396	1.77	1.674	
H	Pine	429	1.82	1.776	yes
H	Pine	437	1.79	1.734	yes
H	Pine	438	1.75	1.671	
H	Pine	452	2.07	1.761	yes
H	Pine	466	1.82	1.837	yes
H	Pine	500	1.77	1.718	
H	Pine	514	1.82	1.693	
L	Pine	22	1.18	1.175	yes
L	Pine	23	1.19	1.275	
L	Pine	50	1.17	1.155	yes
L	Pine	61	1.16	1.162	yes
L	Pine	158	1.23	1.204	yes
L	Pine	177	1.25	1.228	
L	Pine	187	1.2	1.15	yes
L	Pine	209	1.23	1.296	

L	Pine	242	1.22	1.255	
L	Pine	329	1.15	1.343	
L	Pine	331	1.1	1.221	
L	Pine	336	1.19	1.301	
L	Pine	404	1.22	1.294	
L	Pine	422	1.15	1.268	
L	Pine	441	1.09	1.127	yes
L	Pine	443	1.02	1.176	yes
L	Pine	446	1.2	1.331	
L	Pine	453	1.19	1.26	
L	Pine	457	1.15	1.191	yes
L	Pine	459	1.09	1.192	yes
L	Pine	463	1.2	1.233	
L	Pine	464	1.21	1.254	
L	Pine	467	1.11	1.269	
L	Pine	470	1.01	1.18	yes
L	Pine	475	1.22	1.314	
L	Pine	477	1.21	1.289	
L	Pine	478	1.17	1.272	
L	Pine	499	1.11	1.257	
L	Pine	506	1.2	1.277	
H	Warner	7	1.98	1.896	
H	Warner	8	2.03	1.896	
H	Warner	18	2.07	1.87	
H	Warner	22	2.14	1.899	yes
H	Warner	27	2.04	1.785	
H	Warner	29	1.97	1.94	yes
H	Warner	41	2.1	1.92	yes

H	Warner	44	1.95	1.752	
H	Warner	61	2.28	1.845	
H	Warner	67	2.11	1.933	yes
H	Warner	68	2.07	1.935	yes
H	Warner	69	2.12	1.927	yes
H	Warner	73	1.99	1.759	
H	Warner	100	2.05	1.796	
H	Warner	111	2.19	1.869	
H	Warner	115	2.13	1.897	
H	Warner	128	2.02	1.674	
H	Warner	129	2.04	1.872	
H	Warner	137	2.1	1.763	
H	Warner	145	1.96	1.957	yes
H	Warner	148	2	1.852	
H	Warner	160	2.25	2.036	yes
H	Warner	166	2.14	1.916	yes
H	Warner	172	2.03	1.832	
H	Warner	174	2.02	1.76	
H	Warner	178	2.04	1.803	
H	Warner	186	2.16	1.939	yes
H	Warner	188	2.12	1.872	
H	Warner	189	1.96	1.796	
L	Warner	4	1.53	1.369	yes
L	Warner	11	1.52	1.57	yes
L	Warner	16	1.58	1.527	yes
L	Warner	20	1.61	1.701	
L	Warner	33	1.54	1.616	
L	Warner	36	1.44	1.393	yes

L	Warner	39	1.6	1.635	
L	Warner	54	1.52	1.473	yes
L	Warner	56	1.51	1.428	yes
L	Warner	79	1.59	1.564	yes
L	Warner	96	1.55	1.676	
L	Warner	113	1.57	1.636	
L	Warner	121	1.6	1.675	
L	Warner	130	1.58	1.622	
L	Warner	132	1.58	1.71	
L	Warner	143	1.44	1.569	yes
L	Warner	153	1.56	1.626	
L	Warner	200	1.55	1.631	
L	Warner	202	1.58	1.754	
L	Warner	205	1.57	1.495	yes
L	Warner	212	1.58	1.535	yes
L	Warner	222	1.58	1.638	
L	Warner	234	1.47	1.577	

Table S4: Statistical tests of correlated divergence between large- and small-selected lines. Variance indicates whether variances were pooled or kept separate between selection direction, *df* shows the degrees of freedom, *t* gives the test statistic. Traits are as follows: L_4 = length when the 4th clutch was released, L_0 = length at birth, $C_{1\text{-}4}$ = total offspring in the first four clutches, g_Adult = adult specific growth rate, g_Juve = juvenile specific growth rate, *r* = intrinsic rate of increase, A = age at maturity, ICI = inter-clutch interval.

**Population**	**Trait**	**Variance**	**df**	**t-statistic**	**p-value**
Bristol	L_4	separate	12.20	10.1449	**> 0.0001**
	L_0	pooled	17	4.3044	**0.0038**
	$C_{1\text{-}4}$	pooled	17	7.944	**> 0.0001**
	g_Adult	pooled	17	3.6903	**0.0145**
	g_Juve	separate	9.73	0.0882	1.0000
	r	separate	13.32	3.1466	**0.0602**
	A	pooled	17	3.0839	**0.0539**
	ICI	pooled	17	0.2987	1.0000
Little Long	L4	separate	14.42	8.6835	**> 0.0001**
	L_0	pooled	18	3.2359	**0.0367**
	$C_{1\text{-}4}$	separate	13.25	8.6027	**> 0.0001**
	g_Adult	separate	12.39	3.015	**0.0834**
	g_Juve	pooled	18	-0.1035	1.0000
	r	separate	12.89	4.0286	**0.0117**
	A	separate	17.25	0.0933	1.0000
	ICI	pooled	18	0.2622	1.0000
Pine	L_4	pooled	18	9.8183	**> 0.0001**

	L_0	separate	15.23	2.5121	**0.1898**
	C_{1-4}	separate	17.05	6.6888	**> 0.0001**
	g_Adult	separate	15.45	1.1102	1.0000
	g_Juve	pooled	18	1.328	1.0000
	r	separate	17.35	4.3431	**0.0034**
	A	separate	16.94	1.6391	0.9570
	ICI	separate	13.99	-2.6482	0.1528
Warner	L_4	separate	17.24	2.8876	**0.0810**
	L_0	separate	17.17	1.7625	0.7662
	C_{1-4}	separate	13.06	3.9465	**0.0133**
	g_Adult	separate	17.65	1.3424	1.0000
	g_Juve	separate	15.09	0.7098	1.0000
	r	separate	13.74	0.0312	1.0000
	A	separate	14.57	2.1427	0.3956
	ICI	separate	16.19	-0.8911	1.0000

Table S5. Regression analysis showing relationship between size at maturity and other life history traits. Slope indicates the regression coefficient, S.E. gives the standard error of the coefficient. Significance tests are taken all have *df* = 1, 18 (*df* = 1,17 for traits in Bristol) and are Bonferroni corrected for multiple comparisons within populations.

**Population**	**Trait**	**Slope**	**S.E.**	**F-ratio**	***p***
Bristol	L_4	1.4628	0.1558	88.1247	**0.0000**
	L_0	0.1469	0.0344	69.8957	**0.0005**
	C_{1-4}	66.5265	12.0521	30.4696	**0.0000**
	G_Juve	0.0372	0.0280	1.7682	0.2012
	G_Adult	0.0085	0.0070	1.4924	0.2385
	A	1.1347	0.4294	9.8333	**0.0060**
	r	0.2466	0.0696	12.5375	**0.0025**
	ICI	0.1122	0.1635	0.4714	0.5016
Little Long	L_4	1.5221	0.1084	197.2054	**0.0000**
	L_0	0.1360	0.0360	14.2854	**0.0014**
	C_{1-4}	71.7810	6.2534	131.76	**0.0000**
	G_Juve	-0.0047	0.0185	0.0656	0.8007
	G_Adult	0.0168	0.0051	10.9238	**0.0039**
	A	0.2309	0.6331	0.133	0.7196
	r	0.2321	0.0502	21.4134	**0.0002**
	ICI	-0.0514	0.2127	0.0583	0.8119
Pine	L_4	1.1622	0.0934	154.7328	**0.0000**

	L_0	0.0848	0.0321	6.9922	**0.0165**
	C_{1-4}	42.0943	5.2115	65.2412	**0.0000**
	G_Juve	0.0460	0.0394	1.3618	0.2585
	G_Adult	0.0053	0.0080	0.4827	0.4961
	A	1.0441	0.6318	2.7309	0.1158
	r	0.3219	0.0736	19.136	**0.0004**
	ICI	-0.6119	0.2402	6.4893	**0.0202**
Warner	L_4	1.5476	0.1851	69.8957	**0.0000**
	L_0	0.3081	0.0546	31.8235	**0.0000**
	C_{1-4}	59.4050	12.9316	21.103	**0.0000**
	G_Juve	0.0338	0.0216	2.4492	0.1350
	G_Adult	0.0144	0.0082	3.051	0.0977
	A	-0.2761	1.3532	0.0416	0.8406
	r	0.1560	0.1216	1.6478	0.2155
	ICI	-0.2911	0.3282	0.787	0.3867

Table S6. Tests of whether regression slopes in Table S5 differ among populations (i.e., whether a significant interaction between population and size at maturity is evident). For all traits, *df* = 3, 71 for the interaction term.

**Trait**	**F-ratio**	***p***
L_4	1.3260	0.2727
L_0	3.5455	**0.0187**
C_{1-4}	1.6944	0.1760
G_juve	0.8017	0.4971
G_adult	0.5822	0.6286
Age @ maturity	0.9740	0.4099
r	0.5175	0.6716
ICI	1.9210	0.1340

Figure S1: Size distribution of clones in the base populations as measured in the first body size assay. Bars are labeled with the floor of each size class, in mm.

Figure S2: Correlated divergence of clutch size in the first through fourth clutch in clones selected for large (gray bars) or small (white bars) body size. Error bars show standard errors. Note that vertical axes have different scales.

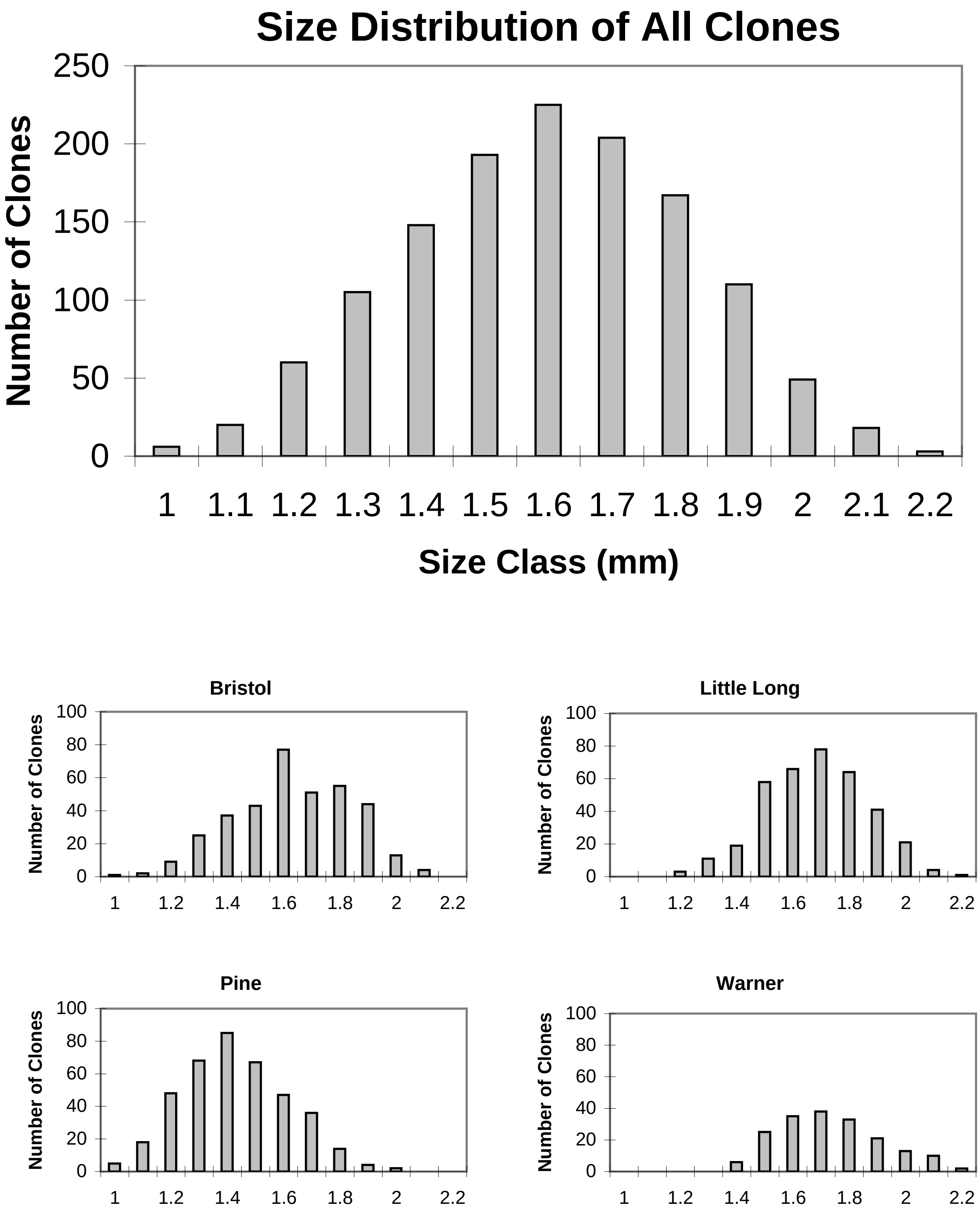

Figure S1: Size distribution of clones as measured in the first body size assay.
Bars are labeled with the floor of each size class, in mm.

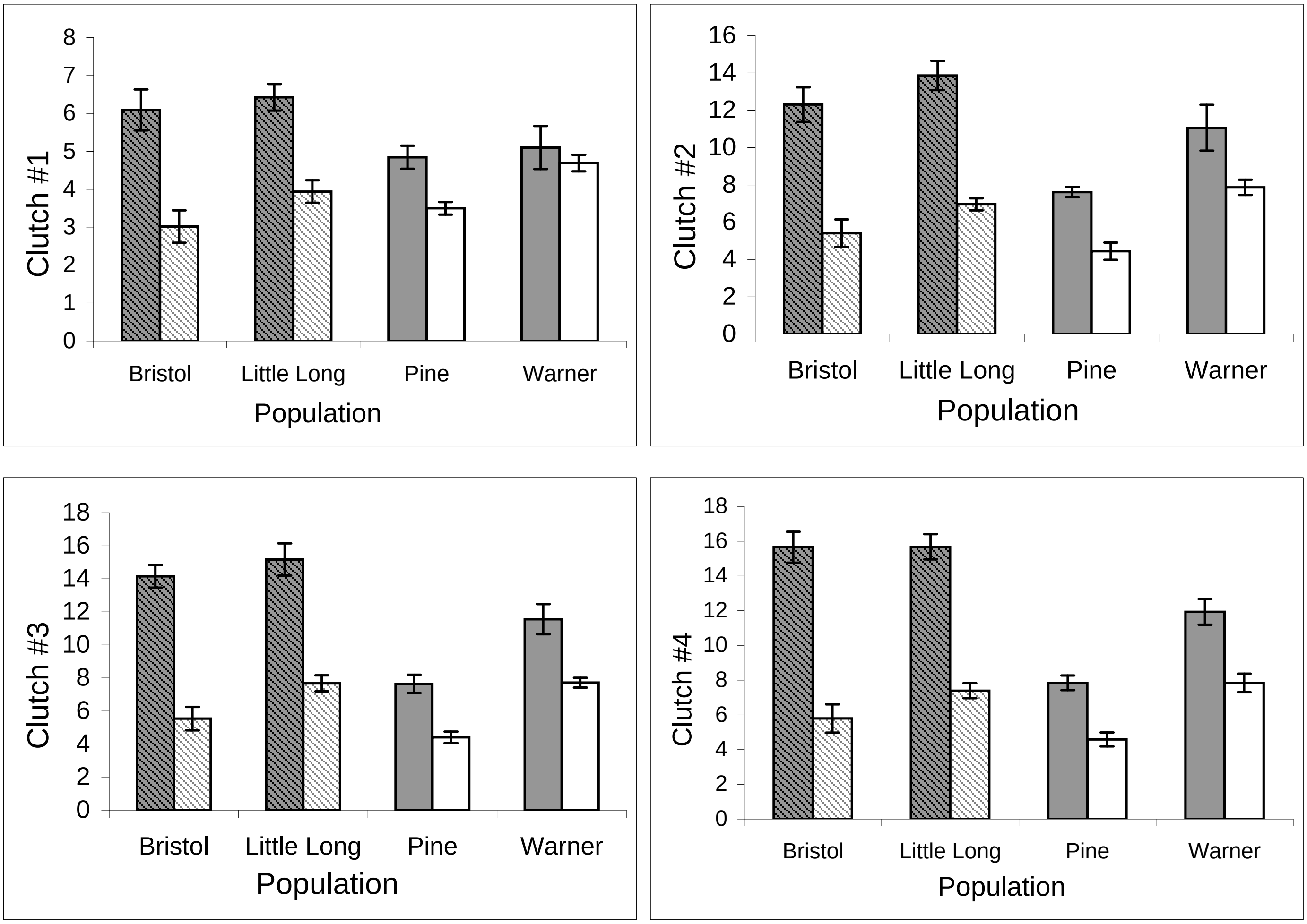
Clutch #1
8
7
6
5
4
3
2
1
0
Bristol
Little Long
Pine
Warner
Population
Clutch #2
16
14
12
10
8
6
4
2
0
Bristol
Little Long
Pine
Warner
Population
Clutch #3
18
16
14
12
10
8
6
4
2
0
Bristol
Little Long
Pine
Warner
Population
Clutch #4
18
16
14
12
10
8
6
4
2
0
Bristol
Little Long
Pine
Warner
Population

Figure S2
